# Endoplasmic reticulum degradation impedes olfactory G-protein coupled receptor functional expression

**DOI:** 10.1186/1471-2121-5-34

**Published:** 2004-09-15

**Authors:** Min Lu, Lena Staszewski, Fernando Echeverri, Hong Xu, Bryan D Moyer

**Affiliations:** 1 Senomyx, Inc., 11099 North Torrey Pines Road, La Jolla, CA 92037, USA; 2 Present Address: Kalypsys, Inc., 10420 Wateridge Circle, San Diego, CA 92121 USA

## Abstract

**Background:**

Research on olfactory G-protein coupled receptors (GPCRs) has been severely impeded by poor functional expression in heterologous systems. Previously, we demonstrated that inefficient olfactory receptor (OR) expression at the plasma membrane is attributable, in part, to degradation of endoplasmic reticulum (ER)-retained ORs by the ubiquitin-proteasome system and sequestration of ORs in ER aggregates that are degraded by autophagy. Thus, experiments were performed to test the hypothesis that attenuation of ER degradation improves OR functional expression in heterologous cells.

**Results:**

To develop means to increase the functional expression of ORs, we devised an approach to measure activation of the mOREG OR (Unigene # Mm.196680; Olfr73) through coupling to an olfactory cyclic nucleotide-gated cation channel (CNG). This system, which utilizes signal transduction machinery coupled to OR activation in native olfactory sensory neurons, was used to demonstrate that degradation, both by the ubiquitin-proteasome system and autophagy, limits mOREG functional expression. The stimulatory effects of proteasome and autophagy inhibitors on mOREG function required export from the ER and trafficking through the biosynthetic pathway.

**Conclusions:**

These findings demonstrate that poor functional expression of mOREG in heterologous cells is improved by blocking proteolysis. Inhibition of ER degradation may improve the function of other ORs and assist future efforts to elucidate the molecular basis of odor discrimination.

## Background

The sense of smell originates in the olfactory epithelium when olfactory receptors (ORs), members of the seven transmembrane domain G-protein coupled receptor (GPCR) superfamily, bind odorant ligands [1,2]. Despite identification of the first constituents of the ~1000 member OR superfamily over a decade ago, efforts to uncover the molecular basis of odor discrimination have been severely limited by the inability to efficiently express ORs at the plasma membrane in heterologous expression systems [2-6].

Recently, we elucidated three specific cellular mechanisms responsible for inefficient OR trafficking to the plasma membrane: ORs are retained within the endoplasmic reticulum (ER) due to inefficient folding and poor coupling to ER export machinery, degraded via the ubiquitin-proteasome system, and sequestered in ER aggregates that are degraded by autophagy [7]. Thus, we have a clearer understanding of the problems associated with OR expression in heterologous cells.

To develop rationale means to improve the functional expression of ORs, an approach was devised to quantitate activation of the mouse mOREG OR (Unigene # Mm.196680; Olfr73), which recognizes the odorant eugenol (spicy, cinnamon-like odor) [8], following coupling to an olfactory cyclic nucleotide-gated cation channel (CNG) [9]. Using this assay, we show that degradation by both the ubiquitin-proteasome system and autophagy limits mOREG functional expression. Our results demonstrate for the first time that inhibition of proteolysis can positively modulate OR function.

## Results and discussion

### Functional expression of mOREG using a CNG-based assay

A cell-based approach was developed to measure functional expression of mOREG in heterologous cells. This system was designed to mimic the signal transduction events involved in OR activation in the olfactory epithelium and utilizes CNG as a cAMP biosensor [10,11]. In olfactory sensory neurons, odorant binding to ORs initiates a signaling cascade involving the heterotrimeric G protein G_olf_, adenylate cyclase III, and an olfactory CNG, leading ultimately to the sensation of smell [1,12]. Accordingly, we transiently co-expressed mOREG, as an N-terminal fusion protein with the first 20 amino acids of rhodopsin (Rho20-mOREG), with untagged olfactory CNG subunits in HEK293 cells that endogenously express both the heterotrimeric G protein G_s_, a functional homologue of G_olf _[13,14], and adenylate cyclase III [15]. The Rho tag has been shown to facilitate chemosensory GPCR functional expression [8,16,17], possibly by enhancing translocation into the ER during protein synthesis. In this system, odorant binding to Rho-mOREG, which couples to endogenous G_s _and elicits increases in the second messenger cAMP in HEK cells [8,18,19], leads to the opening of CNG and the influx of calcium from the extracellular medium. Thus, Rho-mOREG function can be directly correlated with cellular calcium levels.

**Figure 1:**
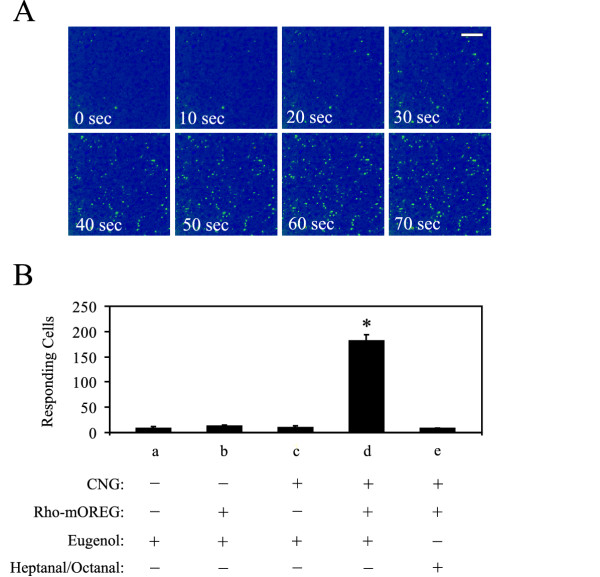
**Functional expression of Rho-mOREG using a CNG-based assay. **(A) HEK293 cells transiently transfected with Rho-mOREG and CNG were assayed for increases in intracellular calcium in response to 100 uM eugenol. Images contain ~2000 confluent cells and represent responses at the indicated times following odorant stimulation. Scale bar is 300 um. Responses were observed in ~10% of total cells or ~20% of transfected cells (~50% transfection efficiency). (B) Quantitation of cells expressing vector only (a), Rho-mOREG only (b), CNG only (c), Rho-mOREG and CNG (d and e) following stimulation with 100 uM eugenol (a-d) or 100 uM heptanal plus 100 uM octanal (e). * p < 0.001 compared to cells expressing vector only (a).

Responses to eugenol, a ligand for mOREG, were detected in cells co-expressing Rho-mOREG and CNG (Fig. 1A and 1B) but not in cells expressing vector only, Rho-mOREG only, or CNG only (Fig. 1B). Eugenol activation of Rho-mOREG became evident at 20 sec and increased to a maximal level at 50 sec following odorant application. The EC_50 _for eugenol (20.8 +/- 3.4 uM) closely approximated published values (35 uM and 46 uM), thereby validating the utility of our approach [8,19]. Rho-mOREG activation was specific for eugenol as no response was observed when cells were challenged with the control odorants heptanal and octanal, which activate the I7 OR (Fig. 1B) [4,16,20]. Collectively, these data demonstrate that Rho-mOREG can functionally couple to CNG in heterologous cells following odorant stimulation.

### ER degradation limits mOREG functional expression

**Figure 2:**
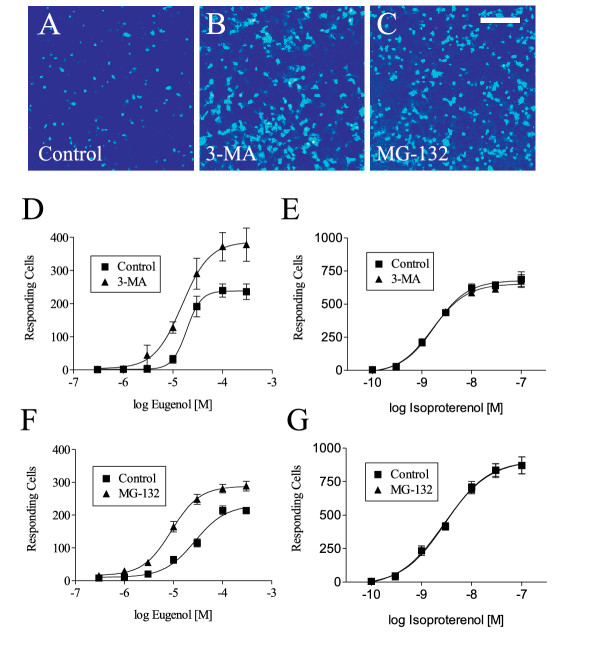
**ER degradation limits Rho-mOREG functional expression. **CNG cells transiently transfected with Rho-mOREG were assayed for increases in intracellular calcium in response to 10 uM eugenol following treatment with vehicle (control, 0.1% DMSO; A), 10 mM 3-MA (B) or 50 uM MG-132 (C) for 4 h. Images contain ~750–1000 confluent cells and represent responses 60 sec following odorant stimulation. Scale bar is 150 um. Dose-response curves were determined for eugenol (300 nM to 300 uM; D and F) or isoproterenol (100 pM to 100 nM; E and G) in cells treated with vehicle (control), 10 mM 3-MA (D and E) or 50 uM MG-132 (F and G) for 4 h. ~20–25% of total cells or ~40–50% of transfected cells (~50% transfection efficiency) responded to 100 uM eugenol under control conditions. Note that degradation inhibitors specifically increased Rho-mOREG functional responses and had no effect on β-AR function. The EC_50 _value for eugenol was larger for control (17.1 +/- 3.0 uM) compared to 3-MA (10.8 +/- 2.6 uM) and MG-132 (8.1 +/- 2.6 uM) treatments, but these differences did not achieve statistical significance. The EC_50 _values for isoproterenol for control (3.7 +/- 1.0 nM), 3-MA (4.2 +/- 2.5 nM), and MG-132 (2.6 +/- 0.9 nM) treatments were not significantly different.

Previously, we demonstrated that ORs are inefficiently expressed at the plasma membrane of heterologous cells due, in part, to degradation of ER-retained ORs by the ubiquitin-proteasome system and sequestration of ORs in ER aggregates that are degraded by autophagy [7]. Specifically, inhibition of the proteasome using MG-132 or inhibition of autophagy using 3-methyladenine (3-MA) enhanced Rho-mOREG protein expression 2 to 3-fold by biochemical and cellular analyses [7]. Therefore, experiments were performed to test the hypothesis that ER degradation limits OR functional expression. These studies used cells stably expressing CNG and transiently expressing Rho-mOREG to achieve more robust responses. As shown in Figure 2, treatment of cells with 3-MA, a specific inhibitor of autophagy that blocks sequestration of material into autophagosomes [21], enhanced Rho-mOREG activation by eugenol (compare Fig. 2A and 2B). Similar results were obtained when cells were treated with MG-132 (compare Fig. 2A and 2C) or epoxomicin (data not shown), a specific inhibitor of the proteasome [22]. Autophagy and proteasome inhibitors increased Rho-mOREG functional expression 2 to 3-fold in the linear range of eugenol dose response curves (Fig. 2D and 2F). Importantly, 3-MA and MG-132 specifically promoted Rho-mOREG function, since responses of the β-adrenergic receptor (β-AR), a GPCR that utilizes the same signal transduction machinery as Rho-mOREG (GPCR-G_s_-adenylate cyclase) [14], following isoproterenol challenge were unaffected (Fig. 2E and 2G). Thus, degradation inhibitors were not modulating the function or trafficking of elements of the signal transduction machinery, including CNG, coupled to Rho-mOREG. Collectively, these data suggest that ER degradation, by autophagy and the ubiquitin-proteasome system, limits Rho-mOREG functional expression.

**Figure 3:**
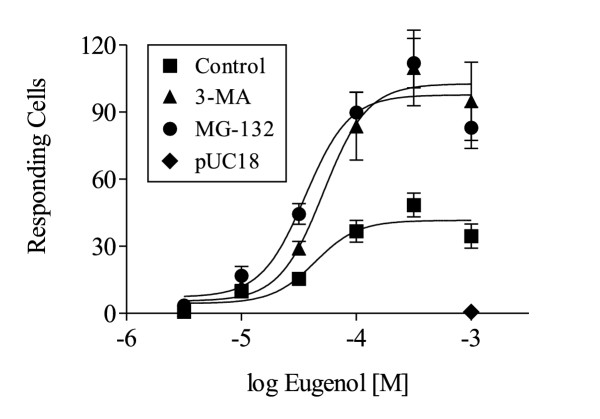
**ER degradation limits untagged mOREG functional expression. **CNG cells transiently transfected with untagged mOREG were assayed for increases in intracellular calcium in response to increasing concentrations of eugenol (from 3 uM to 1000 uM) following treatment with vehicle (control), 10 mM 3-MA, or 50 uM MG-132 for 4 h. The EC_50 _values for eugenol for control (36.5 +/- 12.9 uM), 3-MA (42.6 +/- 6.5 uM), and MG-132 (36.4 +/- 7.5 uM) treatments were not significantly different. CNG cells transfected with vector only (pUC18) and treated with vehicle, 3-MA, or MG-132 did not respond to 1000 uM eugenol.

To determine if increased functional expression of Rho-mOREG following inhibition of ER degradation was attributable to use of an OR fusion protein, we examined the effect of autophagy and proteasome inhibitors on mOREG lacking the Rho tag. Although eugenol activation of Rho-mOREG (EC_50 _= 17.1 +/- 3.0 uM; maximum number of responding cells at 300 uM eugenol = 214 +/- 10) was more robust than untagged mOREG (EC_50 _= 36.5 +/- 12.9 uM; maximum number of responding cells at 300 uM eugenol = 48 +/- 5; p < 0.05 compared to Rho-mOREG for both EC_50 _and maximal number of responding cells), likely due to an established role of the Rho tag in facilitating chemosensory GPCR functional expression [8,16,17], MG-132 and 3-MA increased untagged mOREG functional expression 2 to 3-fold (Fig. 3), similar to the magnitude observed with Rho-mOREG (Fig. 2). Thus, inhibition of ER degradation increased Rho-mOREG and untagged mOREG functional expression, demonstrating that observed effects were not attributable to use of a non-native OR fusion protein.

**Figure 4:**
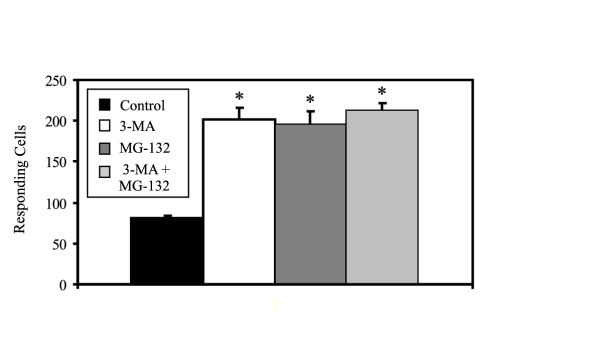
**Simultaneous inhibition of autophagy and the proteasome does not produce additive Rho-mOREG functional responses. **CNG cells transiently transfected with Rho-mOREG were assayed for increases in intracellular calcium in response to 10 uM eugenol following treatment with vehicle (control), 10 mM 3-MA, 50 uM MG-132, or 10 mM 3-MA plus 50 uM MG-132 for 4 h. Individual or combined treatment with degradation inhibitors yielded similar levels of Rho-mOREG activity. * p < 0.001 compared to cells treated with vehicle.

Two seemingly independent mechanisms degrade ORs retained in the ER. First, OR aggregates sequestered in ER subdomains are targeted to lysosomes for degradation by autophagy, and second, misfolded ORs are covalently modified by polyubiquitination and degraded by the proteasome [7]. To determine if simultaneous inhibition of autophagy and the ubiquitin-proteasome system produced additive effects on Rho-mOREG function, we co-treated cells with 3-MA and MG-132. As shown in Figure 4, Rho-mOREG function, measured using 10 uM eugenol, a non-saturating concentration near the EC_50 _value, was equivalent in cells treated with 3-MA alone, MG-132 alone, or 3-MA plus MG-132.

The non-additive effects of 3-MA and MG-132 on Rho-mOREG function suggest one of the following two non-mutually exclusive scenarios: first, OR degradation by autophagy may be linked to OR degradation by the ubiquitin-proteasome system, by a poorly defined mechanism as suggested for other aggregation prone proteins [23-25]; second, in addition to ER degradation, an additional step(s) downstream of proteolysis may limit Rho-mOREG functional expression in heterologous cells. A recent preliminary report described specialized accessory proteins that increase OR surface expression and function [26]. These proteins could serve as chaperones to package OR cargo into COPII vesicles for export from the ER and/or couple ORs to requisite signal transduction machinery at the plasma membrane, steps that are both downstream of ER degradation. In the absence of necessary accessory proteins, functional expression may not exceed a certain level regardless of the quantity of OR that is diverted from the degradative pathways by autophagy and ubiquitin-proteasome inhibitors. The existence of multiple steps limiting OR functional expression in heterologous cells is further supported by our findings that the function of mOREG, lacking the Rho tag, is less robust than Rho-mOREG. Since the Rho tag may facilitate translocation into the ER during protein synthesis [8,16,17], ER translocation could comprise an additional limiting step, upstream of ER degradation, for OR functional expression.

### ER export and trafficking through the Golgi apparatus are necessary for Rho-mOREG functional expression

Inhibition of ER degradation events could permit a pool of Rho-mOREG to achieve an ER export competent conformation and traffic through the Golgi apparatus to the plasma membrane. However, using surface biotinylation, surface immunofluorescence microscopy, flow cytometry, and glycosidase digestion assays, we were unable to demonstrate convincing Rho-mOREG surface expression or visualize a pool of Rho-mOREG containing endoglycosidase H-resistant carbohydrate modifications indicative of transit through the Golgi apparatus following treatment with ER degradation inhibitors (ML and BDM unpublished observations). These results suggested that either a small pool of properly folded Rho-mOREG was expressed at the plasma membrane in quantities below the threshold of the cell biological techniques employed to visualize the receptor, or that a pool of intracellular Rho-mOREG comprised the functionally responsive population in calcium imaging experiments. Notably, numerous studies have documented the functional expression of GPCRs and requisite signal transduction machinery in intracellular compartments, including ER membranes [27-29].

To differentiate between these two possibilities, we adopted a pharmacological approach to selectively and independently block trafficking from compartments in the early secretory pathway, specifically the ER and Golgi apparatus. If inhibition of ER degradation does not increase Rho-mOREG activity under conditions that block export from ER and Golgi compartments, the functionally responsive Rho-mOREG population is likely derived from an internal pool that is required to traffic to the plasma membrane to function. Conversely, if inhibition of ER degradation increases Rho-mOREG activity under conditions that block export from ER and Golgi compartments, the functionally responsive Rho-mOREG population likely resides in an intracellular compartment.

**Figure 5:**
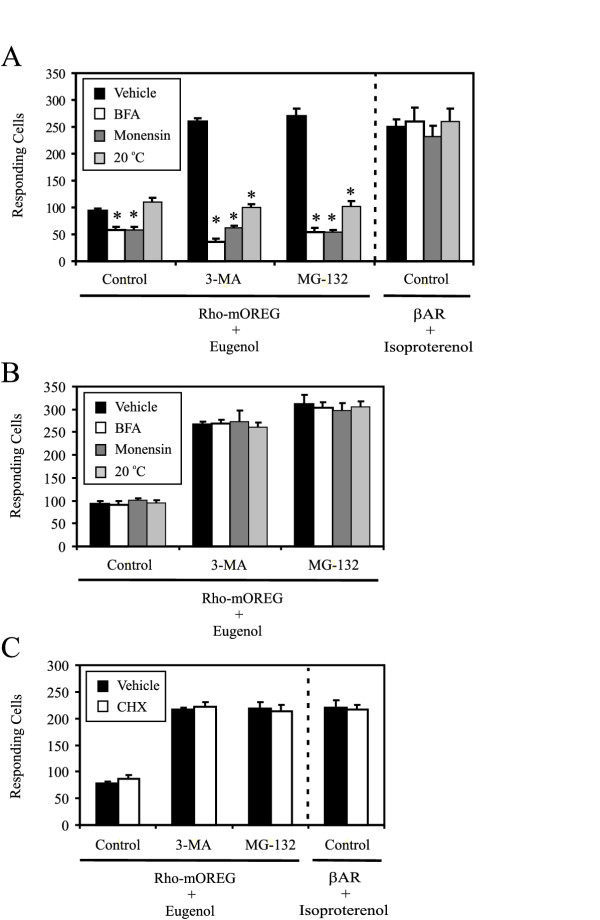
**ER export and trafficking through the Golgi apparatus are required for Rho-mOREG functional expression. **CNG cells transiently transfected with Rho-mOREG were assayed for increases in intracellular calcium in response to 10 uM eugenol, to gauge Rho-mOREG function, or 1 nM isoproterenol, to gauge β-AR function, following treatment with vehicle (0.1% ethanol), BFA (5 ug/ml), monensin (10 uM), or incubation at 20°C for 4 h (A) or 5 min (B). (C) Cells were treated with vehicle or CHX (75 uM) for 4 hr. In all experiments, cells were also co-treated for 4 h with control (0.1% DMSO), 10 mM 3-MA, or 50 uM MG-132 as indicated. Partial inhibition of Rho-mOREG function with BFA or monensin but not 20°C (A, control columns), a temperature that also attenuates endocytic events, is likely attributable to turnover of cell surface Rho-mOREG when the biosynthetic pathway, which normally replenishes the plasma membrane Rho-mOREG pool, is blocked. By contrast, BFA and monensin do not affect function of the β-AR, a control GPCR that exhibits a long half-life at the plasma membrane [31]. * p < 0.005 compared to cells treated with vehicle in the same group.

To inhibit protein trafficking from the ER, brefeldin A (BFA), which blocks ER export of cargo proteins by inducing collapse of the Golgi stacks into the ER, was used [30]. As shown in Fig. 5A, BFA completely blocked the enhancement of Rho-mOREG function by 3-MA and MG-132; by contrast, BFA had no effect on functional responses of the β-AR, indicating that BFA was not affecting the function or trafficking of signal transduction machinery, including CNG, coupled to Rho-mOREG. By blocking transport of proteins present in the ER that are in route to the plasma membrane, specifically Rho-mOREG following inhibition of ER degradation, BFA inhibited Rho-mOREG function; β-AR and CNG function were unperturbed since these proteins were already present at the plasma membrane prior to BFA treatment. Since β-AR exhibits a long half-life at the plasma membrane [31], inhibiting delivery of newly synthesized β-AR by BFA would not adversely affect isoproterenol responses. Thus, ER export is required for increased Rho-mOREG functional expression by degradation inhibitors.

To inhibit protein trafficking from the Golgi apparatus, monensin, an ionophore that disrupts Golgi structure and inhibits Golgi trafficking events, was used [32]. Monensin, similar to BFA, completely blocked the enhancement of Rho-mOREG function by 3-MA and MG-132 while having no effect on β-AR function (Fig. 5A). Similar effects were observed when cells were incubated at 20°C (Fig. 5A), a temperature that arrests protein transport at trans Golgi cisternae [33]. Importantly, trafficking disrupting agents did not affect Rho-mOREG or β-AR function when acutely applied to cells, further substantiating that results were not attributable to non-specific effects on signal transduction machinery or eugenol binding (Fig. 5B). Thus, Golgi trafficking events are required for increased Rho-mOREG functional expression by degradation inhibitors.

Inhibition of Rho-mOREG function by trafficking disrupting agents, specifically BFA, could be due to activation of the unfolded protein response [34] and inhibition of Rho-mOREG protein synthesis. To directly address this possibility, experiments were performed to test the effect of the protein synthesis inhibitor cycloheximide (CHX) on functional expression of Rho-mOREG. As shown in Figure 5C, CHX, used at concentrations previously demonstrated to inhibit Rho-mOREG translation [7], had no effect on increased Rho-mOREG function by 3-MA and MG-132. These data suggest that autophagy and ubiquitin-proteasome inhibitors diverted an existing pool of Rho-mOREG from degradative pathways to the secretory pathway and that effects of degradation inhibitors and trafficking disrupting agents were not attributable to modulation of protein synthesis.

Collectively, these data support a model whereby inhibition of ER degradation promotes a small fraction of Rho-mOREG to achieve an ER export competent conformation, thereby satisfying ER quality control processes, and traffic through the secretory pathway to the plasma membrane. Thus, similar to chemical and pharmacological chaperones that promote folding and restore ER export of misfolded GPCR cargo [35-37], agents interfering with ER degradation may promote ER export of GPCR and non-GPCR cargo [38], trafficking through the biosynthetic pathway, and functional expression at the cell surface. We speculate that the pool of Rho-mOREG expressed at the plasma membrane is below the detection limits of cell biological techniques used to visualize the receptor [7] but above the detection threshold for calcium imaging methodology used to examine receptor function. Indeed, following pharmacological treatments, improved ΔF508 cystic fibrosis transmembrane conductance regulator functional expression at the plasma membrane is readily detectable by sensitive electrophysiological analyses but neither by cell surface labeling nor by biochemical approaches measuring carbohydrate modifications indicative of transit through the Golgi apparatus [39-41]. Though we favor a model whereby inhibition of ER degradation promotes OR export from the ER and improves OR surface expression, we were unable to obtain cellular and biochemical evidence to support this proposal. We cannot exclude the possibility that inhibition of ER degradation also stabilizes an otherwise labile cofactor or chaperone protein, endogenously expressed by heterologous cells, that modulates OR trafficking in a post-Golgi compartment, stability at the cell surface, and/or function at the plasma membrane [42,43].

## Conclusions

We have developed an expression system for ORs that utilizes signal transduction machinery coupled to OR activation in native olfactory sensory neurons. Using CNG as a cAMP biosensor to gauge mOREG function, we demonstrate that inhibition of ER degradation, by both autophagy and the ubiquitin-proteasome system, promotes functional expression of Rho-mOREG as well as untagged mOREG. Thus, proteolysis limits mOREG function in heterologous cells. Inhibition of ER degradation may improve the function of other ORs and assist future efforts to elucidate the molecular basis of odor discrimination.

## Methods

### Molecular biology

Rho20-mOREG expression vector was generated in pRK5 as previously described [7]. To generate untagged mOREG expression vector, mOREG coding sequence was excised using AscI/NotI and subcloned into a modified pRK5 vector lacking the Rho20 tag. The human CNGA2 and CNGB1b expression constructs encode untagged human CNGA2 and CNGB1b in pEAK10-derived vectors (Edge Biosystems, Gaithersburg, MD) [44]. For the generation of stable transfectants, CNGA2 was subcloned into pCDNA3.1/zeo (Invitrogen, Carlsbad, CA). The CNGA2 clone contains the C458W and E581M mutations, introduced using the QuickChange Site-Directed Mutagenesis Kit (Stratagene, La Jolla, CA), previously shown to increase cAMP sensitivity in rat CNGA2 [11].

### Compounds, odorants, and ligands

BFA, isoproterenol, monensin, and 3-MA were from Sigma (St. Louis, MO); MG-132 was from Calbiochem (San Diego, CA); eugenol, heptanal and octanal were from Aldrich (Milwaukee, WI).

### Cell culture and transfections

HEK293 cells were maintained and transfected as previously described [7]. For the generation of CNG stable transfectants, cells were transfected with linearized CNGA2 and CNGB1b expression constructs (1:1 ratio) and selected using 50 ug/mL zeocin (Invitrogen) and 0.5 ug/mL puromycin (Calbiochem). Individual colonies were expanded and screened for CNG expression by assaying functional responses to 500 uM eugenol following transient transfection with Rho-mOREG. For functional expression studies, CNG cells were grown in media without selection 72 h prior to experimentation and transiently transfected with mOREG 48 h prior to experimentation.

### mOREG functional expression

Functional expression of mOREG was investigated using calcium imaging methodology as previously described [44]. Cells seeded in 24 well plates were loaded with the calcium dye fluo-4 acetoxymethyl ester (Molecular Probes, Eugene, OR) 2 d post-transfection using the following conditions: 3 uM dye in 0.5 ml Hanks' balanced salt solution containing divalent cations (HBSS, Invitrogen) for 1 h at room temperature in the dark. Cells were subsequently washed once with 0.5 ml HBSS to remove excess fluo-4, supplemented with 0.25 ml HBSS, and then stimulated with an additional 0.25 ml HBSS containing the appropriate ligand at twice the final concentration. A single ligand was applied to each dish of cells. Typically, 3–4 separate dishes of cells were used for each ligand concentration or condition and experiments were repeated 3–4 times. Thus, individual data points represent the average of 9–16 separate measurements.

Changes in intracellular calcium were monitored by fluorescence microscopy using an Axiovert S100 TV inverted microscope with a 10× long working distance Plan Fluor objective (numerical aperture 0.5) and a cooled charge-coupled device camera (Princeton Instruments, Trenton, NJ). Images were acquired using a Lambda DG-4 automated wavelength controller (Sutter Instrument Co., Novato, CA) at 480 nm excitation and 535 nm emission and analyzed using Imaging Workbench 4.0 (Axon Instruments, Union City, CA). Counting the number of cells responding to ligands 60 sec following stimulus addition, when cells had achieved a maximal response, quantitated receptor activity. This established methodology has been used to functionally characterize the human T1R1/T1R3 umami receptor, the human T1R2/T1R3 sweet receptor, and the Drosophila Gr5a trehalose receptor [44,45]. To independently validate this method, we determined that the EC_50 _for isoproterenol activation of the β-AR (3.7 +/- 1.0 nM), measured by counting responding cells, closely matched published values (1.7–3.3 nM), measured either by fluorescent intensity measurements or a cAMP accumulation assay [10]. In addition, the EC_50 _for eugenol activation of Rho-mOREG (20.8 +/- 3.4 uM), measured by counting responding cells, closely approximated the published value (35 uM and 46 uM), determined by monitoring fluorescent intensity of responding cells [8,19]. Finally, the EC50s for glutamate activation of mGluR4 and cycloheximide activation of mT2R05 were similar when determined by counting cells or by monitoring fluorescent intensity [44].

We estimate ~40–50% of transfected CNG cells express functional cell surface Rho-mOREG based on the following points. First, ~20–25% of total cells in a microscopic field respond to maximal doses of eugenol. Second, ~50% of cells are transfected, measured by either co-transfection with red fluorescent protein or by immunolabelling permeabilized cells expressing Rho-mOREG with an anti-Rho antibody. Thus, ~40–50% of transfected cells express sufficient Rho-mOREG at the plasma membrane to elicit a functional response.

### Statistics

Data represent the mean +/- SEM. Statistical significance was determined using an unpaired, two-tailed Student's t-test. Dose-response curves were plotted and EC_50 _values were determined using GraphPad Prism v3.02 software.

## Authors' contributions

ML generated the mOREG expression vectors and carried out most of the calcium imaging experiments. LS generated the cells stably transfected with CNG. FE assisted with the calcium imaging experiments. HX generated the CNG expression vectors. BDM coordinated the study and wrote the paper.
